# High‐frequency oscillations after acute hemorrhagic traumatic brain injury: insights into posttraumatic epilepsy development

**DOI:** 10.1002/epi.70235

**Published:** 2026-06-02

**Authors:** Kseniia Kriukova, Lin Li, Richard J. Staba, Misque Boswell, Tuba Asifriyaz, Rachel Thomas, James J. Gugger, Mohamad Shamas, Ramon Diaz‐Arrastia, Manuel Buitrago Blanco, Jerome Engel, Paul M. Vespa, Dominique Duncan, the EpiBios4Rx investigators, Denes Agoston, Alicia K. Au, Michael Bell, Ben Churn, Jan Claassen, Ramon Diaz‐Arrastia, Jerome Engel, Brandon Foreman, Aristea Galanopoulou, Emily Gilmore, Martin Hunn, Nathalie Jette, Andrew Morokoff, Solomon L. Moshé, Terence O'Brien, Joshua Laing, Piero Perucca, Kristine H. O'Phelan, Asla Pitkanen, Eric Rosenthal, Frederick Willyerd, Lara Zimmermann, Ben Ellingson, Buitrago Blanco, Daniel Correa, Dana Harrar, Thomas P. Bleck, Brian Appavu, Aaron Struck, Baxter Allen, Inna Keselman, Jeff Kennedy, Victor Ferastraoaru, Ji Yeoun Yoo, Arthur W. Toga

**Affiliations:** ^1^ Department of Neurology Perelman School of Medicine, University of Pennsylvania Philadelphia Pennsylvania USA; ^2^ Department of Neurology David Geffen School of Medicine, University of California, Los Angeles Los Angeles California USA; ^3^ Department of Biomedical Engineering University of North Texas Dallas Texas USA; ^4^ Department of Neurology Barrow Neurological Institute Phoenix Arizona USA; ^5^ Department of Neurology, School of Medicine and Dentistry University of Rochester Rochester New York USA; ^6^ Brain Research Institute University of California, Los Angeles Los Angeles California USA; ^7^ Department of Neurosurgery David Geffen School of Medicine, University of California, Los Angeles Los Angeles California USA; ^8^ Department of Neurobiology David Geffen School of Medicine, University of California, Los Angeles Los Angeles California USA; ^9^ Department of Psychiatry and Biobehavioral Sciences David Geffen School of Medicine, University of California, Los Angeles Los Angeles California USA

**Keywords:** epileptogenesis, high‐frequency oscillations, posttraumatic epilepsy, traumatic brain injury

## Abstract

**Objective:**

The development of posttraumatic epilepsy after traumatic brain injury (TBI) is potentially identifiable by measuring biomarkers of epileptogenesis, namely pathological high‐frequency oscillations (pHFOs). pHFOs are promising candidates, but it remains uncertain whether they can be detected early after TBI in clinical settings. This study was undertaken to determine the incidence and location of pHFOs as recorded from scalp and intracranial electroencephalography (EEG) during the first week after acute TBI and to determine the association of pHFOs with late posttraumatic seizures (PTS).

**Methods:**

We analyzed continuous EEG from 35 TBI patients with acute hemorrhagic TBI (Glasgow Coma Scale = 3–13) enrolled in the multicenter EpiBioS4Rx cohort. Automated pHFO detection was followed by independent experts' verification. The rate of two types of pHFO ripples (70–250 Hz) and fast ripples (250–500 Hz) were computed using scalp and intracranial EEG. Firth logistic regression models estimated associations between pHFOs rates and late PTS occurrence.

**Results:**

16 of 35 patients (45.7%) developed late PTS. Verified scalp ripples were observed in 17 patients (48.6%), whereas no fast ripples were confirmed on expert review. Ripple activity was most frequent over frontal regions (13/17, 76.5%, *p* = .049, 95% confidence interval [CI] = .50–.93) and similarly frequent in perihemorrhagic locations (13/17, 76.5%, *p* = .049, 95% CI = .50–.93). Among 10 patients with intracranial EEG, verified ripples occurred in five (50%), including two of two with pericontusional strip electrodes.

**Significance:**

In critically ill patients with acute hemorrhagic TBI monitored in the intensive care unit, both scalp and intracranial EEG can detect pHFOs within the first week after injury, demonstrating the technical feasibility of capturing these signals in a real‐world clinical setting. Verified pHFOs were most frequently observed over frontal and perihemorrhagic regions, consistent with early perilesional hyperexcitability. pHFOs appear to be mechanistically grounded markers of perilesional hyperexcitability. Standardized, high‐sampling EEG studies with targeted pericontusional coverage are needed to establish prognostic performance for late PTS.

**Key points**

- This is the first study to systematically examine pHFOs and late PTS in acute hemorrhagic TBI in clinical settings.
- pHFOs are detectable via both scalp and intracranial continuous EEG during acute hemorrhagic TBI in the ICU settings.
- Verified pHFOs were most frequently observed over frontal and perihemorrhagic regions, consistent with preclinical findings.
- No fast ripples were expert‐verified, likely reflecting ICU artifact burden and sampling limitations.

## 1 INTRODUCTION

Traumatic brain injury (TBI) represents a significant global health burden, marked by high mortality rates and long‐term disabilities.^1^, ^2^ Among its complications, posttraumatic epilepsy (PTE) is a significant long‐term disability with impact on quality of life.^3^, ^4^, ^5^ PTE is defined by the onset of unprovoked late posttraumatic seizures (PTS) occurring more than 7 days after TBI, whereas seizures occurring within the first 7 days (early PTS) are considered acute symptomatic seizures.^6^, ^7^, ^8^, ^9^, ^10^ After severe TBI, a single late PTS meets the International League Against Epilepsy criteria for epilepsy due to the high risk of recurrent seizures.^11^, ^12^, ^13^, ^14^ Therefore, we use “late PTS” and “PTE” interchangeably in this analysis.

PTE represents 20% of symptomatic epilepsy cases in the general population and accounts for 5% of all epilepsy patients attending specialized centers.^3^, ^15^, ^16^, ^17^, ^18^ Notably, approximately 80% of individuals with PTE experience their first seizure within the first 12 months postinjury, and more than 90% have seizures by the end of the second year.^8^, ^19^, ^20^ Moreover, existing studies suggest that PTE can develop years after the initial trauma, underscoring the extended risk and the necessity for continuous monitoring and intervention in these patients.^21^, ^22^, ^23^ Identifying which TBI survivors will progress to PTE therefore remains a pressing clinical goal, and would significantly facilitate the design of clinical trials of antiepileptogenic therapies. Although numerous biomarker candidates spanning biofluid analytes, genomic profiles, advanced imaging, and neurophysiological measures have been proposed, none has yet met the thresholds for clinical implementation.^24^ Electroencephalography (EEG) is inexpensive, noninvasive, and deployable at the bedside, making it an attractive platform for discovering such prognostic signatures.^24^, ^25^ Although a substantial number of groups have tested potential EEG‐based predictors,^24^, ^26^, ^27^, ^28^ to date, none has been clinically validated for this purpose.

In recent years, pathologic high‐frequency oscillations (pHFOs) have been extensively studied for their role in epileptogenesis.^29^, ^30^, ^31^, ^32^ pHFOs are defined as distinct EEG events comprising at least four oscillations that are distinguishable from the background EEG activity.^30^, ^32^, ^33^, ^34^ Most groups classify pHFOs into ripples (80–250 Hz) and fast ripples (250–500 Hz).^35^, ^36^ Also, some of the groups proposed a lower edge for the clinical HFOs (>70 Hz).^37^, ^38^, ^39^ These oscillations can be either pathological (pHFOs) or physiological and may arise spontaneously or be induced.^40^, ^41^ Physiological HFOs (also termed nonpathological HFOs) are high‐frequency oscillatory events generated by normal brain activity and are not associated with epileptic pathology. In contrast, pHFOs are linked to disease states, most notably epilepsy, and frequently localize to epileptogenic zones.^29^, ^30^, ^42^, ^43^, ^44^, ^45^ In practice, these subtypes are often differentiated based on recording location; HFOs detected within the seizure onset zone or early spread zone are classified as pathological, whereas those found outside these epileptogenic areas, specifically in regions without interictal spikes or seizures, are considered physiological. Also, it is essential to note that both types represent the same general phenomenon (fast oscillatory bursts), and they can occur in the same patient or brain region under different conditions.^33^, ^46^ Following this logic, we hypothesize that pHFOs identified at the site of early PTS in an hemorrhagic TBI survivor or recorded from pericontusional brain tissue represent pHFOs.

Extensive animal modeling indicates that pHFOs, as well as repetitive HFOs and spikes, might be correlated with the development of PTE.^47^, ^48^, ^49^, ^50^

To date, and to the best of our knowledge, these findings have not been investigated in human studies in acute clinical settings,^51^ highlighting a significant gap between animal research and human studies. Most studies dedicated to exploring pHFOs in humans are conducted on patients who already have epilepsy, whereas there is still a largely unexplored area in studying those at high risk of developing the condition who have not yet done so, such as patients with acute hemorrhagic TBI.

In this study, we aim to investigate the detectability and characteristics of pHFOs, including ripples (70–250 Hz), and in cases with sufficient sampling rate, fast ripples (250 Hz‐500 Hz), during the first week posttrauma in both intracranial and scalp EEG in patients with acute hemorrhagic TBI. We also examine the relationship between HFOs and the subsequent development of late PTS.

## 2 MATERIALS AND METHODS

This work is part of the EpiBioS4Rx study, a multicenter, longitudinal epilepsy bioinformatics study aimed at developing antiepileptogenic therapies.^52^ Enrollment criteria included acute hemorrhagic TBI patients with evidence of intracranial bleeding (subdural, subarachnoid, and/or intraparenchymal) on computer tomography (CT), aged 6–100 years, Glasgow Coma Scale (GCS) scores of 3–13 without continuous sedation, and the ability to enroll within 72 h of injury. Exclusion criteria included the following: diffuse axonal injury without hemorrhagic contusions or skull fractures; isolated epidural hemorrhages that improved postevacuation; lack of a plan for continuous EEG (cEEG) monitoring from injury days 1–7; inability to undergo magnetic resonance imaging (MRI) from postinjury days 1–18; known conditions such as human immunodeficiency virus/acquired immunodeficiency syndrome and hepatitis B or C; pregnancy; preexisting neurological diseases such as TBI, stroke, or neurodegenerative disorders; preexisting central nervous system malignancy; preexisting epilepsy or seizure disorders; preexisting dementia; isolated anoxic brain injury; severe cervical spine injury; brain death; incarceration (current or pending); the inability of the patient or family to commit to the study until day 730; a positive severe acute respiratory syndrome coronavirus 2 test; or any other condition or circumstance deemed by the investigator to potentially compromise the safety of the participant or the integrity of the study.^52^

cEEG, including surface electrodes and, in selected cases when clinically justified, intracranial monitoring with depth or subdural strip electrodes (mostly linear electrocorticography strip), was performed as part of standard care at each site. Intracranial electrodes were placed at the discretion of the treating neurosurgical team, either on intact brain tissue (subdural strip electrode was placed on the cerebral cortex away from a primary injury or a depth electrode inserted into intact parenchyma) or on pericontusional brain (subdural strip electrode placed on the cerebral cortex close to a primary injury). For scalp recordings, either metal or plastic disk electrodes or needle scalp electrodes, or suction cup electrodes, were arranged in a referential montage. The types of electrodes used in each case and their locations are detailed in Tables 1 and 2. The number of scalp electrodes varied, with a minimum of 12 placed according to the 10–20 system. Upon admission and throughout consideration for enrollment in the EpiBioS4Rx study, cEEG monitoring followed standard practices, typically beginning within 72 h of injury and continuing for 72 h to 1 week.

**TABLE 1:** Overview of scalp high‐frequency oscillations.

No.	Sampling rate, Hz	Type of electrodes	Duration, h	Channels with verified ripples presence	Imaging‐defined tissue type at ripple‐positive electrode pairs	Total ripple rate	Total fast ripple rate	1st review ripple rate	1st review fast ripple rate	Verified ripple rate
1	2048	Scalp needle electrodes	2.93	Cz‐Pz	Edematous	2.557	.536	.073	.049	.024
2	2048	Scalp needle electrodes	1.97	–	–	1.235	.073	.000	.000	.000
3	2048	Metal or plastic disk electrodes	1.82	–	–	7.313	1.219	.118	.354	.000
4	2048	Metal or plastic disk electrodes	.8	–	–	7.411	.268	.000	.000	.000
5	2000	Scalp needle electrodes	1.23	–	–	18.938	1.622	.116	.000	.000
6	500 & 2000	Scalp needle electrodes	3.87	Fp1‐F7	Perihemorrhagic	.776	.000	.018	.000	.018
7	2000	Scalp needle electrodes	1.2	–	–	.833	.060	.060	.000	.000
8	2000	Scalp needle electrodes	1.8	–	–	4.904	.783	.082	.000	.000
9	2000	Scalp needle electrodes	1.97	–	–	11.404	2.034	.254	.036	.000
10	2000	Metal or plastic disk electrodes	.4	–	–	3.214	.000	.000	.000	.000
11	1024	Metal or plastic disk electrodes	.95	–	–	3.051	.000	.000	.000	.000
12	512	Metal or plastic disk electrodes	.95	Fp1‐F7, C3‐P3	Perihemorrhagic	28.571	–	.376	–	.150
13	500	Metal or plastic disk electrodes	1.85	Fp2‐F8	Perihemorrhagic	1.415	–	.039	–	.039
14	512	Metal or plastic disk electrodes	1.65	–	–	21.390	–	.232	–	.000
15	512	Metal or plastic disk electrodes	3.6	Fp1‐F7	Perihemorrhagic	10.216	–	.087	–	.087
16	512	Metal or plastic disk electrodes	2.54	Fp1‐F7	Perihemorrhagic	1.349	–	.040	–	.040
17	512	Metal or plastic disk electrodes	1.05	Fp1‐F7, F7‐T3, F4‐C4, F3‐C3	Perihemorrhagic	19.878	–	.113	–	.113
18	512	Metal or plastic disk electrodes	1.24	–	–	15.510	–	.000	–	.000
19	512	Metal or plastic disk electrodes	.4	Fp1‐F7	Perihemorrhagic	11.988	–	.347	–	.058
20	512	Scalp needle electrodes	3.64	Fp2‐F8, F4‐C4, T5‐O1	Perihemorrhagic, edematous	43.714	–	1.429	–	1.143
21	500	Scalp needle electrodes	2.74	–	–	4.954	–	.000	–	.000
22	500	Scalp needle electrodes	3.67	T3‐T5, T5‐O1	Edematous	45.967	–	.392	–	.131
23	512	Suction cup electrodes	2.67	F8T4, T5‐O1, T4‐T6	Perihemorrhagic, edematous	50.223	–	.348	–	.214
24	512	Scalp needle electrodes	.9	Fp1‐F7, F7‐T3, F8T4, T4‐T6, T6‐O2	Perihemorrhagic, edematous	60.670	–	.509	–	.214
25	512 & 2000	Scalp needle electrodes	1.85	F4‐C4, C3‐P3, T6O2	Perihemorrhagic, edematous	149.444	.000	.714	.000	.238
26	512	Suction cup electrodes	1.94	–		.116	–	.000	–	.000
27	512	Metal or plastic disk electrodes	3.04	T6O2	Normal‐appearing	2.365	–	.111	–	.037
28	512	Metal or plastic disk electrodes	2.94	F4‐C4, F3‐C3	Perihemorrhagic	39.890	–	.188	–	.071
29	500	Metal or plastic disk electrodes	.8	F4‐C4, F3‐C3	Perihemorrhagic	3.453	–	.049	–	.049
30	1024	Metal or plastic disk electrodes	.95	C4‐P4	Normal‐appearing	10.268	.000	.357	.000	.089
31	1024	Metal or plastic disk electrodes	1.95	–	–	1.429	.226	.000	.000	.000
32	500	Metal or plastic disk electrodes	1.82	–	–	.696	–	.000	–	.000
33	500	Metal or plastic disk electrodes	.43	–	–	7.551	–	.204	–	.000
34	1024	Metal or plastic disk electrodes	.8	–	–	.517	.000	.000	.000	.000
35	512	Metal or plastic disk electrodes	1.19	–	–	31.684	–	.000	–	.000

**TABLE 2:** Overview of intracranial high‐frequency oscillations.

No.	Samplin rate, Hz	Type of electrodes	iEEG electrode location, hemisphere	iEEG electrode location, tissue	Duration, h	Total ripple rate	Total fast ripple rate	1st review ripple rate	1st review fast ripple rate	Verified ripple rate
1	2048	Depth	Left	Normal	1.0	3.055	1.017	1.017	0	1.013
3	2048	Strip	Right	Pericontusional	1.82	84.954	16.883	3.119	.183	1.103
6	2000	Depth	Left	Normal	1.85	2.308	.427	.000	.000	.000
8	2000	NA	NA	NA	.8	1.057	.288	.000	.000	.000
9	2000	Depth	Right	Normal	1.97	.000	.000	.000	.000	.000
12	512	Strip	Right	Pericontusional	.95	33.684	–	1.316	–	.789
13	500	Strip	Right	Normal	1.78	3.578	–	.367	–	.367
14	512	Strip	Right	Normal	1.65	51.486	–	.675	–	.405
25	512	Depth	NA	NA	.9	.000	–	.000	–	.000
26	2000	Depth	Left	Normal	1.85	.000	.000	.000	.000	.000

Abbreviation: NA, not available.

**FIGURE 1:**
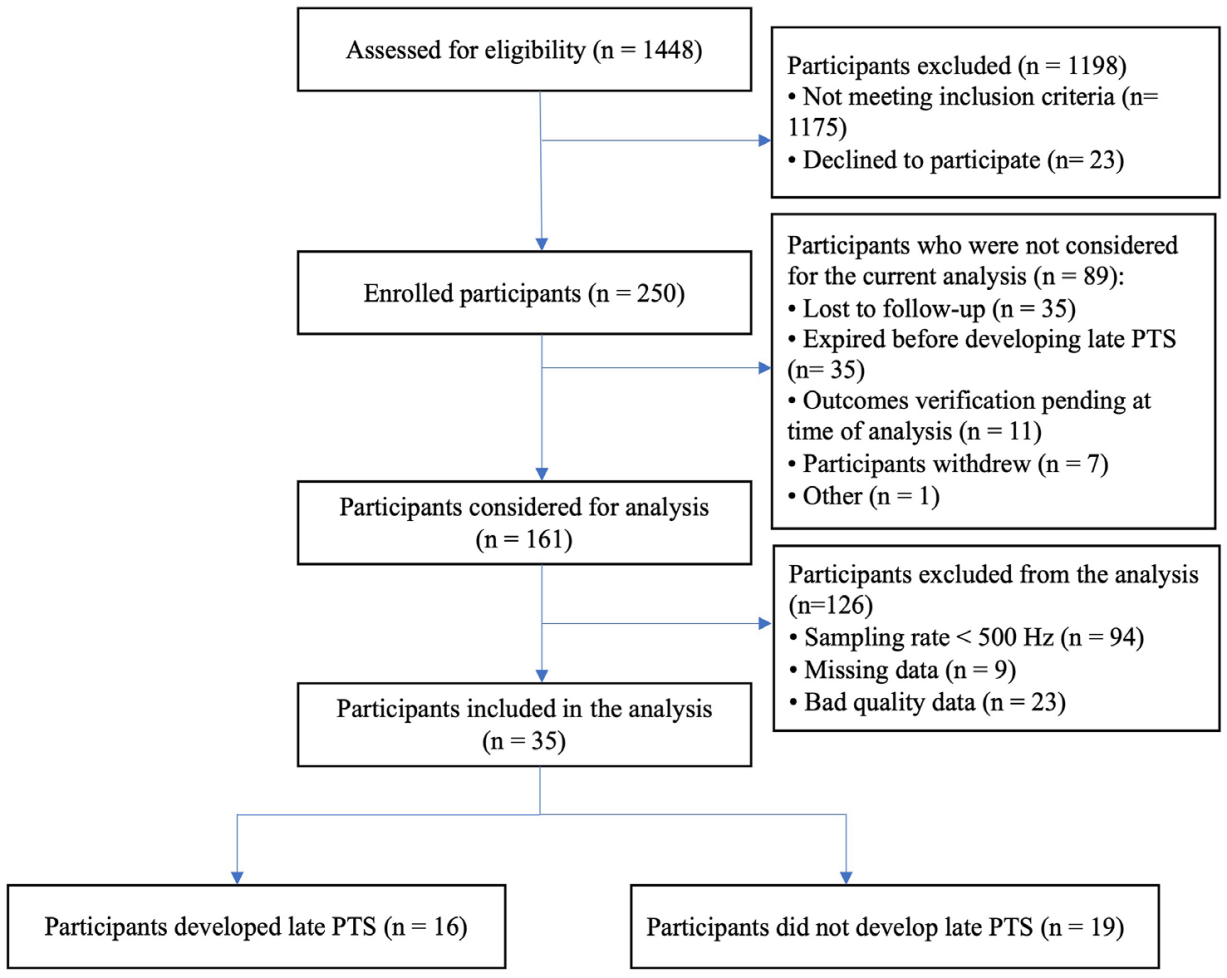
Study flow diagram. PTS, posttraumatic seizures.

A total of 161 participants were initially considered for this analysis. These participants either completed a 2‐year follow‐up without developing seizures or developed late PTS at least 1 week after TBI. In cases where late PTS developed, completing the 2‐year follow‐up was not considered necessary, as it was already confirmed that the patients had developed late PTS. Of these, 126 participants were excluded for various reasons summarized in the study flow diagram (Figure 1). The diagram was adapted from the CONSORT format.^53^ A total of 35 participants had usable EEG sampled at >500 Hz and were included in the ripple analyses (*n* = 10 at 2048 Hz, *n* = 5 at 1024 Hz, *n* = 20 at 512 Hz). The fast ripple subset (sampling rate >1000 Hz) included 15 participants (*n* = 10 at 2048 Hz, *n* = 5 at 1024 Hz), which is detailed in Table 1. Intracranial EEG (iEEG) was available for 10 participants in the ripple cohort and six participants in the fast ripple cohort, which is detailed in Table 2.

Initial visual cEEG analysis was performed using EDFbrowser. The data were initially filtered with the following parameters: high‐pass filter frequency at 1 Hz (following 70‐Hz high‐pass filtering applied during the compilation of preprocessing for further HFO detection), low‐pass filter at 250 Hz for ripple analysis, and 500 Hz for fast ripple analysis, and a notch filter at 60 Hz and its harmonics (finite impulse response filter) using Fieldtrip functions.^54^ The data were rereferenced using the bipolar longitudinal anteroposterior montage. The following electrode pairs were analyzed: Fz‐Cz, Cz‐Pz, F3‐C3, C3‐P3, F4‐C4, C4‐P4, Fp1‐F7, Fp2‐F8, F7‐T3, F8‐T4, T3‐T5, T4‐T6, T5‐O1, and T6‐O2. HFOs on the intracranial electrodes were assessed at the referential montage (as recorded). Although iEEG for HFO analysis is often reviewed with bipolar referencing to reduce far‐field and white matter contamination, we analyzed our iEEG in the original referential montage, because coverage was sparse and reliable adjacent pairs were frequently unavailable; this approach is consistent with earlier studies that used a referential montage.^55^, ^56^, ^57^ Bad channels with poor signal quality were identified and removed.

Given the limited amount of EEG sampled at ≥500 Hz, we tried to preserve as much data as possible; therefore, when standard filtering was insufficient and noisy epochs were too long to excise, we used EEGLAB (MATLAB) to perform independent component analysis with second‐order blind identification (SOBI) to attenuate artifacts.^58^ Because HFO detection is sensitive to preprocessing, SOBI was applied only when necessary for severe contamination. We did not prospectively log the exact segments to which SOBI was applied; based on study notes, it was used in fewer than 13% of segments. Residual artifacts were removed by manual visual inspection after filtering and, when applicable, after SOBI.

**FIGURE 2:**
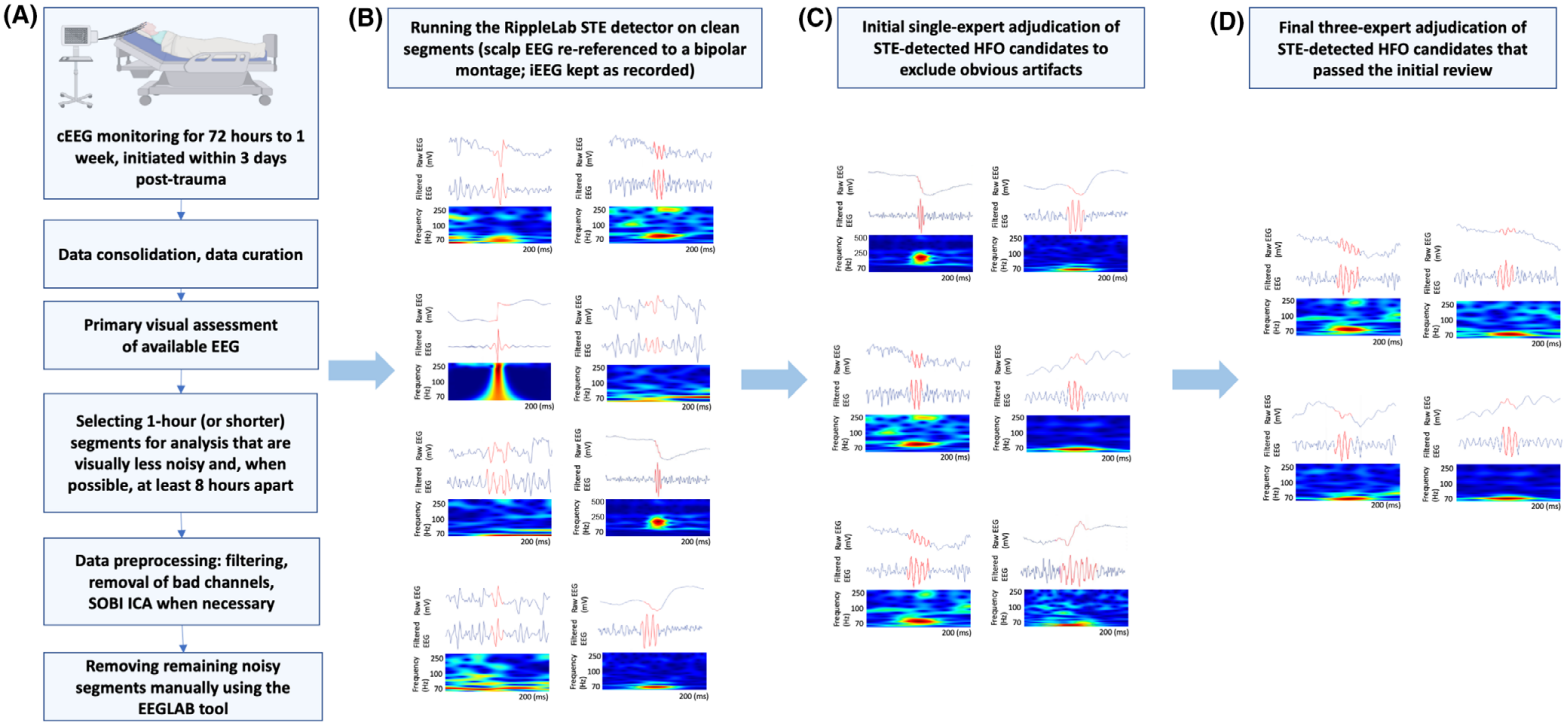
Workflow for continuous electroencephalographic (cEEG) curation and high‐frequency oscillation (HFO) detection/adjudication (patient image in panel A was created with BioRender https://BioRender.com/b0kb871).^63^ (A) Data intake and preprocessing steps. (B) Automated detection using the RippleLab short‐time energy (STE) detector on clean segments (scalp EEG rereferenced to a bipolar montage; intracranial EEG [iEEG] kept as recorded). (C) Initial single‐expert adjudication of STE‐detected HFO candidates to exclude obvious artifacts. (D) Final three‐expert adjudication of candidates who passed the initial review. In all example panels, the upper trace shows the raw (unfiltered) EEG, and the lower panel shows the 70–250‐Hz (70–500‐Hz) time–frequency representation over a 200‐ms window centered on the HFO peak (highlighted in red). ICA, independent component analysis; SOBI, second‐order blind identification.

HFOs were detected with the short‐time energy (STE) method^59^ as implemented in the MATLAB‐based RippleLab toolbox.^60^ Detector settings were as follows: root mean square (RMS) window = 3 ms, detection threshold > 2 SD above the channelwise RMS baseline, peak threshold > 3 SD above the mean of the band‐passed signal, minimum duration = 20 ms with ≥4 oscillations; events separated by <10 ms were merged. We used a lower band‐pass cutoff of 70 Hz (rather than 80 Hz) to increase scalp sensitivity,^61^ RMS window 3 ms to preserve brief oscillatory bursts, and thresholds of 2 SD (RMS) and 3 SD (peak) to favor sensitivity. We constrained morphology with a minimum duration of 20 ms and ≥4 oscillations and merged events separated by <10 ms, balancing sensitivity with specificity. All detections then underwent two‐stage visual review: an initial screen (1st review) by K.K., followed by adjudication by a three‐expert panel (R.J.S., L.L., K.K.), which mitigated the higher false‐positive risk associated with the sensitive parameterization. Each expert rated the events as follows: 1 = definite verified (true) event, 2 = questionable, and 3 = artifact. These ratings were summed and categorized as follows: 3–4 points = verified (true) event (Category 1), 5–7 points = questionable (Category 2), and ≥8 points = artifact (Category 3). Only events labeled as Category 1 were considered to have survived the review. Overall interrater agreement was 69.1%; kappa = .41 (95% confidence interval [CI] = .31–.52).^62^ Because the RippleLab settings were tuned to favor sensitivity while maintaining reasonable specificity, and to ensure transparency, we report three rates: the raw RippleLab detection rate (total ripple and total fast ripple rates), the rate after the initial screen by K.K. (first review ripples and first review fast ripple rates), and the rate after adjudication by the three‐expert panel (R.J.S., L.L., K.K.; verified ripple rate). The full processing pipeline is shown in Figure 2.

### 2.1 Statistical analysis

For each participant, we calculated ripple and fast ripple rates in three categories—total detections (ripples and fast ripples), first‐review detections (ripples and fast ripples), and verified detections (ripples only, because no fast ripple detections survived review by three experts)—using only analyzable scalp EEG channels. Rates per hour were obtained according to the following formula:

$$\text{Ripple Rate}\;\left(\text{Fast Ripple Rate}\right)/h=60\times \frac{\text{Total Events}\;\left(n\;\text{Ripples or}\;n\;\text{Fast Ripples}\right)}{\begin{array}{c} \text{Channels Minutes} \end{array}}$$

We fit Firth logistic regressions adjusting for GCS. Information on sedative medication dosing and timing was not fully available, so GCS scores measured closest to the EEG recording were used as the best available adjustment for neurological status.

In addition to continuous burden, we examined binary exposures for each ripple definition: total ripples—high versus low, using the full‐sample median rate per hour as the cut‐point; first‐review ripples—present versus absent (≥1 event); and verified ripples—present versus absent (≥1 event).

For each definition, we computed Fisher exact test and then fit Firth logistic models adjusted for GCS, as described above. Because the rate already incorporates exposure time, no additional offset for total channel‐minutes was applied. Wald 95% CIs are reported for the penalized estimates. Primary analyses were restricted to participants aged ≥13 years (adolescents and adults) to minimize physiological heterogeneity between pediatric and adult EEGs. A sensitivity analysis including the single 7‐year‐old participant produced the same qualitative conclusions.

All statistical tests were two‐sided with *α* = .05. When multiple comparisons were conducted, *p*‐values were adjusted using the Benjamini–Hochberg false discovery rate (FDR) procedure, and adjusted *q*‐values are reported where applicable. Analyses were performed in R (2024.04.2+764) using the packages tidyverse, stringr, lubridate, logistf, irr, and dplyr.^64^, ^65^

## 3 RESULTS

Of 35 patients, 16 (45.7%) developed late PTS and six (17%) developed early PTS (Table 3). Most patients were male (77%). A detailed description of the cohort is provided in Table 3. Among the 17 patients who had verified ripples detected, 13 (76.5%) showed frontal involvement (*p* = .049; 95% CI = .50–.93). Likewise, 13 of 17 (*p* = .049, 95% CI = .50–.93) had perihemorrhagic involvement, suggesting that verified ripples most frequently originated from perihemorrhagic regions.

**TABLE 3:** Patients' demographic and clinical characteristics.

No.	Sex	Age, years	PID^a^	GCS^b^	Early PTS	Late PTS	Time of first late PTS occurrence, postinjury	Lesions
1	M	29	2, 5	7, 6	N	Y	3 months–6 months	R Fr SAH; R Fr, R T ICH; R Fr, R T HC
2	M	36	1	9	Y	Y	7 days–1 month	R Fr & R Par EAH; R Fr & R Par aSDH; R Fr & R Par SAH; R Fr & R Par ICH; HC in R T
3	F	68	1	7	Y	Y	1 month–3 months	L&R T EAH; L&R Fr aSDH; L&R SAH; L&R ICH; L&R T HC
4	F	38	7	3	N	Y	1 year–2 years	L&R, L T EAH; L&R Fr, aSDH; L&R Fr SAH; L&R Fr, L T HC
5	M	65	0	11	Y	Y	Day 7–1 month	R T aSDH; R Fr, L Par SAH; L&R Fr, R T ICH; R Fr HC
6	M	24	1, 4	6, 3	N	Y	1 year–2 years	L&R Fr, R Par, R T EDH; R Fr aSDH; R Fr ICH; R Fr HC
7	M	22	3	3	N	N	–	L&R Fr HC
8	M	54	4	9	N	N	–	L Fr, L&R T ICH; L&R T HC
9	M	33	1	3	N	Y	1 year–2 years	L Fr aSDH; L&R Fr, L&R Par SAH; L&R Fr HC
10	M	65	1	8	N	N	–	L&R Fr; L Par, L&R T SAH; L T HC
11	M	40	2	5	N	N	–	R Fr, R Par, R T EAH; R Fr, R Par, R T aSDH; R Fr SAH
12	M	46	3	5	N	Y	1 month–3 months	R Fr EDH; R Fr EAH; R Fr, R T SAH
13	F	7	2	6	N	N	–	L&R Fr aSDH; L Par, L T SAH; L&R Fr, L&R T ICH; L&R Fr, L&R Par, L&R T, L&R O HC
14	M	16	3	3	N	N	–	L T EAH; L T aSDH; L&R Fr, L&R Par, L&R T SAH; L T ICH; L T HC
15	F	62	0, 1	3	N	Y	3 months–6 months	L&R Fr EAH; R Fr aSDH; R Par SAH; R T ICH; R Par, R T HC
16	M	13	1, 2	3	N	N	–	L Fr SAH; L&R Fr HC
17	M	17	1, 2	13	N	N	–	L&R Fr HC
18	M	31	3	3	N	N	–	R Fr, R Par, R T EAH; R Fr, R Par, R T aSDH; R Fr, R T SAH; R Fr ICH; L&R Fr, R T HC
19	M	58	2	13	N	N	–	L&R Fr, R Par, R T EAH; R Fr, R Par, R T aSDH; L&R Fr, R T SAH; R T ICH; L&R Fr, L&R T HC
20	M	53	1	3	Y	Y	1 year–2 years	R Fr, R Par, R T SAH; R T ICH; R T HC
21	M	22	1, 2	3	N	N	–	R Fr, R Par, R T aSDH; R Fr, R Par, R T SAH
22	M	68	1, 2	9	N	N	–	R Fr, L Tent aSDH; L Fr HC
23	M	47	1, 2	3	N	N	–	L&R T EAH; L&R T aSDH; L&R Fr, L Par, L&R T SAH; L Fr HC
24	M	70	1, 2	8	N	Y	1 year–2 years	L Fr SAH; L&R Fr ICH; L&R Fr HC
25	F	62	1	13	N	N	–	L&R Fr aSDH; L&R Fr SAH; L T HC
26	F	22	5	8	N	N	–	L Fr, L T HC
27	F	31	2	3	N	Y	1 year–2 years	L&R Fr aSDH
28	M	66	1, 2	8	N	N	–	L&R T aSDH; L&R Fr HC
29	M	18	0, 1	12	N	Y	1 year–2 years	L&R Fr, R Par, R T EAH; R Fr, R Par, R T aSDH; R Fr SAH; R Fr, R T HC
30	M	15	2	3	N	Y	3 months–6 months	R Fr, R T EDH; R Fr, R T EAH; R Fr, R T aSDH; R Fr, R T SAH; R T ICH; R T & Thal HC
31	M	15	1	3	N	Y	1 year–2 years	L&R T EDH; L&R T EAH; L&R Fr, L Par aSDH; R T SAH; L T ICH; L&R T HC
32	M	40	1, 2	5	N	Y	6 months–1 year	L&R Tent EAH; L Fr, L T aSDH; L&R Fr, L&R T ICH; L&R Fr, L&R T HC
33	F	77	3	4	Y	N	–	L&R Fr aSDH
34	M	72	2	3	Y	N	–	L&R Fr HC
35	M	58	1, 2	7, 9	N	N	–	L&R Fr HC, L&R Fr aSDH, L&R Fr SAH, R Fr ICH

Abbreviations: aSDH, acute subdural hematoma; EAH, extra‐axial hematoma; EDH, epidural hematoma; EEG, electroencephalography; F, female; Fr, frontal; GCS, Glasgow Coma Scale; HC, hemorrhagic contusion; ICH, intracerebral hemorrhage; L, left; L&R, left and right; M, male; N, no; O, occipital; Par, parietal; PID, postinjury day; PTS, posttraumatic seizures; R, right; SAH, subarachnoid hemorrhage; T, temporal; Tent, tentorial; Thal, thalamus; Y, yes.

^a^ PID when analyzed EEG was recorded.

^b^ GCS on the day of analyzed EEG recording.

**FIGURE 3:**
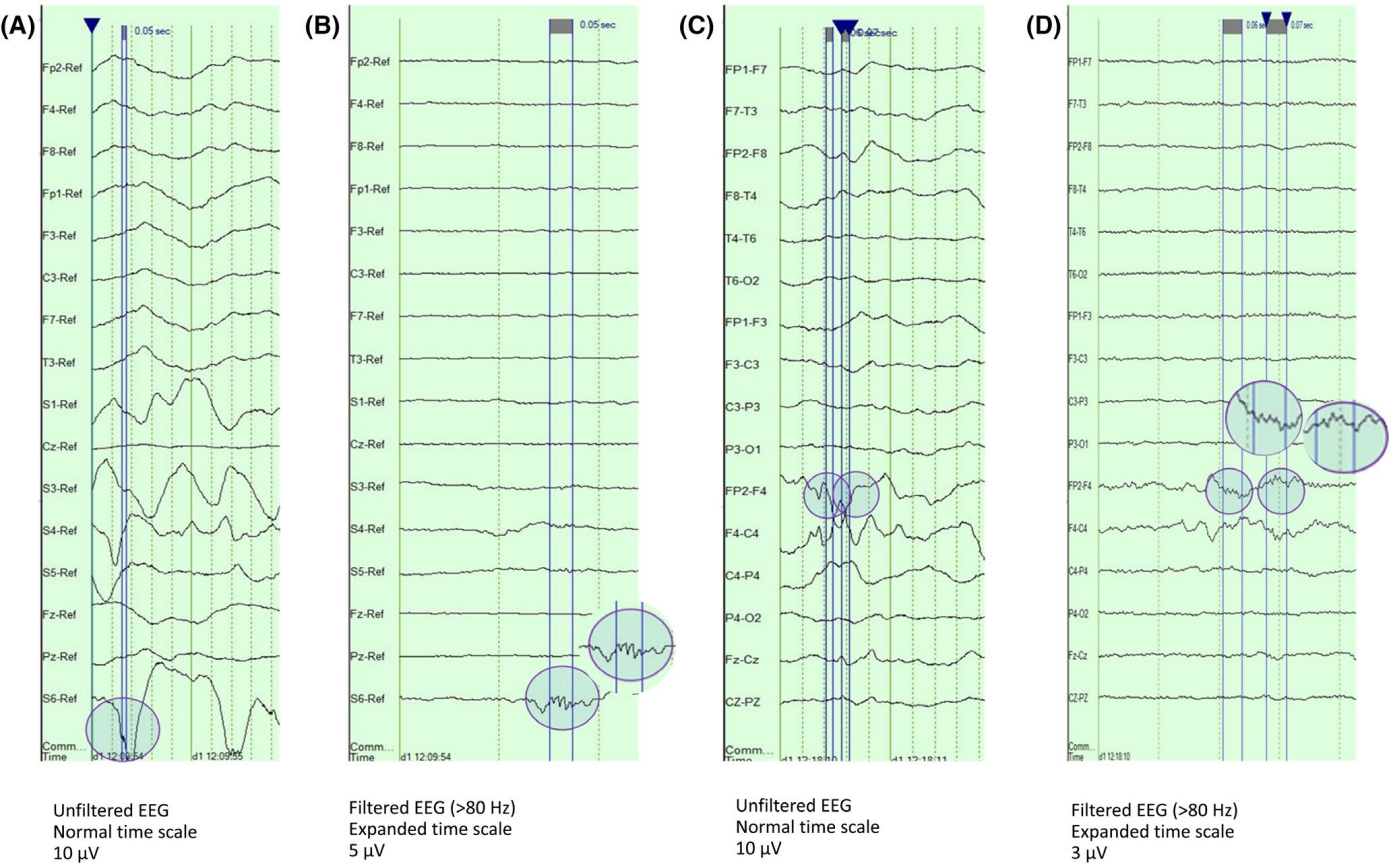
High‐frequency oscillations (HFOs) in intracranial and scalp continuous electroencephalography (EEG), using bipolar montage for scalp EEG and referential (as recorded) for intracranial EEG (iEEG; visualized using Persyst). (A, B)^67^ Referential montage showing both scalp and intracranial electrodes. (A) Unfiltered EEG at normal time scale (10 μV). Scalp EEG is displayed here as referential montage as well for illustrative purposes only, because it is on the same page with iEEG. (B) High‐pass filtered (>80 Hz) EEG at expanded time scale (5 μV), highlighting HFOs in the intracranial electrode. (C, D) Bipolar longitudinal anteroposterior montage (scalp only), focusing on scalp HFOs. (C) Unfiltered EEG at normal time scale (10 μV). (D) High‐pass filtered (>80 Hz) EEG at expanded time scale (3 μV), highlighting HFOs in the scalp electrode. Blue vertical lines and purple circles indicate the temporal and spatial localization of HFO events.

Among those who developed late PTS (*n* = 16), nine (56.3%) showed verified ripple activity; among those without late PTS (*n* = 19), eight (42.1%) had ripples. An example of verified ripples detected on cEEG is presented in Figure 3.

**FIGURE 4:**
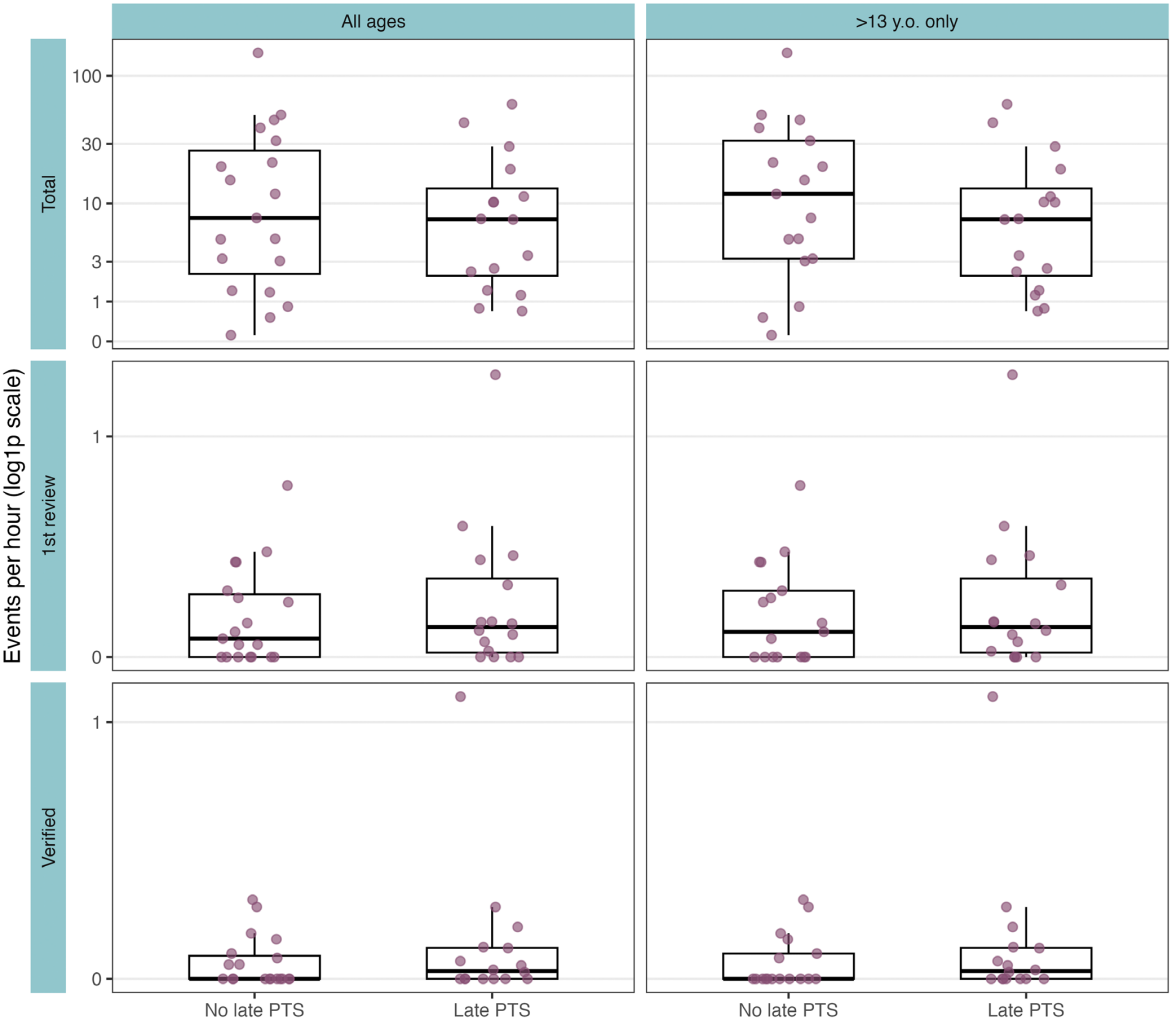
Distribution of scalp ripple rates by late posttraumatic seizure (PTS) status. y.o., years old.

In the surface scalp EEG cohort, total ripple exposure was dichotomized high versus low by the within‐sample median rate (cut = 7.36/h). Overall, 24 of 35 (68.6%) had ≥1 ripple on first review, and 17 of 35 (48.6%) had ≥1 verified ripple. Among those who developed late PTS (*n* = 16), 12 of 16 (75%) had first‐review ripples and nine of 16 (56.3%) had verified ripples; among those without late PTS (*n* = 19), the corresponding proportions were 12 of 19 (63%) and eight of 19 (42%). Comparisons of per‐hour ripple rates between late PTS and non‐late PTS groups showed no significant differences (*p* > .05) (Figure 4).

In Firth logistic regression models adjusting for GCS, fast ripple burden was modestly associated with late PTS (*p* = .028, odds ratio [OR] = 1.16, 95% CI = 1.01–1.38), whereas ripple burden was not (*p* = .78, OR = .96, 95% CI = .73–1.27). After FDR correction across the two continuous exposures, the fast ripple finding was borderline (*q* ≈ .056). Presence‐based definitions (median split, first‐review present/absent, verified present/absent) were not associated with late PTS in GCS‐adjusted Firth models, and corresponding Fisher exact tests were also nonsignificant. Verified scalp ripple presence did not differ by electrode type (cup/disk 10/21, 47.6%; needle 6/12, 50.0%; suction 1/2, 50%; *p* = .486). Verified ripple rates (events per channel‐hour) were also not different across types (*p* = .409).

**FIGURE 5:**
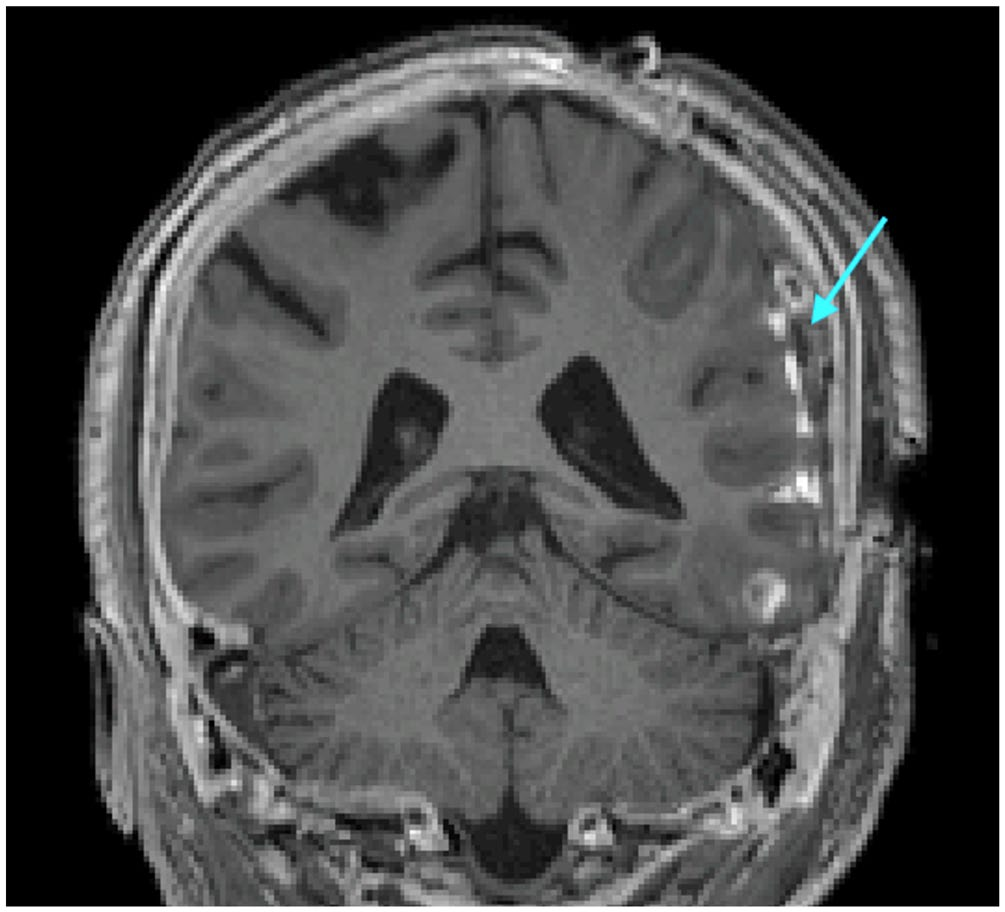
Coronal slice of the preimplant T1‐weighted magnetic resonance imaging coregistered with the postimplant computed tomography, demonstrating a visible implanted intracranial subdural electrode contact (arrow).

Among the 10 patients with usable iEEG, electrode location was available for nine patients; five had depth electrodes (predominantly in radiographically normal tissue) and four had strip electrodes, two of which were placed over pericontusional cortex. A representative coronal slice of the preimplant T1‐weighted MRI coregistered with the postimplant CT, demonstrating a visible implanted strip electrode as shown in Figure 5. To better visualize the location, iEEG coregistration of CT and MRI was performed via iEEG‐recon pipeline.^66^ Both patients with pericontusional strip placement exhibited verified ripples and developed late PTS. In total, verified ripples were detected in five of 10 (50%) iEEG patients. Late PTS occurred in five of 10 (50%) patients, three of whom also had verified ripples (Table 2).

## 4 DISCUSSION

In this exploratory feasibility study, pHFOs were detectable in acute hemorrhagic TBI on both scalp and intracranial cEEG, recorded during the first week posttrauma. Approximately half of the cohort had pHFOs detected, and verified scalp ripple rates were low. Taken together, these observations support the feasibility of monitoring pHFOs acutely and motivate their incorporation into future antiepileptogenesis trials.

We explored the question of tissue type involved in the localization of the pHFOs. In our study, pHFOs occurred mostly in the frontal regions; 13 of 17 (76.5%) participants with ≥1 verified scalp ripple showed frontal involvement (*p* = .049, 95% CI = .50–.93). These areas corresponded to primary hemorrhagic injury locations. Qualitative CT/MRI review indicated that verified scalp pHFOs often colocalized with perihemorrhagic cortex (13/17, 76.5%). Notably, in two patients with subdural strip electrodes placed over pericontusional cortex, pHFOs were observed, and both developed late PTS. These patterns are consistent with preclinical TBI studies in which pHFOs are concentrated in perilesional (perihemorrhagic) cortex.^47^, ^48^, ^68^, ^69^ Although our findings are in agreement with the preclinical data, our results likely underestimate this localization‐dependent relationship due to differences in methodology between animal studies, with electrodes located closely to contusions, and the human EEG with more imprecise and more distantly located electrodes. It is important to note that prior research on EEG correlates of posttraumatic epileptogenesis identified pHFOs, defined in their study as events with central frequencies between 100 and 600 Hz, as being associated with PTE.^47^ Li et al.^68^ showed that although fast ripples provided the stronger predictive marker of PTE, ripples were also significantly more frequent in animals that developed late seizures compared with those that did not. Hence, studying ripples in terms of posttraumatic epileptogenesis is as important as studying fast ripples.

We highlight the importance of pHFO verification in our study. We used visual confirmation of pHFOs and found fast ripples were not confirmed by the three‐expert review in any participant, either on scalp or iEEG. The absence of verified fast ripples stands in contrast to experimental models, where both ripples and fast ripples have been more closely linked to epileptogenesis.^68^, ^69^ Given the technical challenges and the very limited number of participants with sampling rates sufficient for fast ripple analysis, it remains possible that their absence partly reflects our acquisition constraints; fast ripple analysis requires at least ≥1000‐Hz sampling and careful antialiasing/filtering in an artifact‐prone intensive care unit (ICU) environment.^70^, ^71^, ^72^, ^73^ Nevertheless, the lack of verified fast ripples on iEEG is not entirely unexpected in light of our coverage; only six participants had iEEG sampled at ≥1000 Hz, just one with a subdural strip, and the remainder with depth electrodes. Moreover, iEEG fast ripples tend to be spatially restricted to epileptogenic/perilesional tissue, so sampling predominantly normal‐appearing cortex (as in our series) can yield few or no fast ripples.^40^, ^57^ Taken together, the discrepancy between preclinical findings, where fast ripples (250–500 Hz) are associated with posttraumatic epileptogenesis, and our clinical results likely reflects the challenges of high‐frequency EEG analysis in the ICU and the clinical heterogeneity of TBI (lesion type, extent, and location) and iEEG electrode placement/coverage, all of which may obscure or dilute any association between HFOs and late PTS risk.^16^, ^72^, ^74^

Notably, in our study total fast ripple rate based on the original STE detector output showed some trend toward positive association with late PTS (*p* = .028, *q* = .056, OR = 1.16, 95% CI = 1.01–1.38); however, no verified fast ripples were identified. Verification of HFOs remains an active topic of discussion. A growing trend is to rely on automatic detections for scalability and reproducibility. At the same time, these approaches assume limited artifact burden and well‐tuned parameters, and the field still lacks consensus on HFO definitions for different species and within one, a gold‐standard detector or settings, the number of experts required for adjudication, and acceptable interrater agreement thresholds. In addition, convening multiple experts is time‐ and resource‐intensive and may, depending on sampling and case mix, inadvertently bias which events are ultimately retained.

Against this backdrop, we reported all three ripple metrics to make the sensitivity/specificity trade‐off explicit. Detector‐only (total) rates maximize sensitivity but can admit artifacts and sharp transients, especially in ICU recordings even after extensive cleaning. First‐review rates (single‐reader screen) remove obvious false positives while retaining physiologically plausible events. Consensus‐verified rates (three‐expert adjudication) emphasize specificity and face validity, at the cost of fewer events and lower statistical power. This hierarchy is reflected in our data; 24 of 35 (68.6%) participants had ≥1 ripple after the first screen, whereas 17 of 35 (48.6%) remained positive after consensus, approximately 71% of screen‐positive participants. Presence‐based associations with late PTS were directionally similar but nonsignificant at all three stringencies, consistent with either a true null or, more likely, attenuation from measurement error and limited power. In general, nondifferential misclassification biases effects toward the null; moving from detector‐only to verified events should reduce that bias but also reduces information. The observed pattern—ORs > 1 with broad, overlapping CIs across definitions—therefore fits with underpowered detection of small effects, rather than a simple “more stringent = stronger association” expectation.

Regarding comparison of scalp versus iEEG for the detection of pHFOs in our study, in our cohort, iEEG data with adequate sampling rates were available for 10 patients. Similar to scalp recordings, verified ripples were detected in 50% of the cohort. Notably, the majority of iEEG electrodes (approximately 80%) were positioned over radiographically normal‐appearing tissue, whereas both patients with subdural strip electrodes placed over pericontusional cortex exhibited verified ripples and subsequently developed late PTS. Visually, recordings obtained with scalp needle electrodes required substantially less artifact cleaning compared with those recorded using cup or disk electrodes. However, statistical analyses revealed no significant differences between electrode types in either verified ripple rates or ripple presence. Despite the lack of statistical significance, the consistently cleaner signal quality observed with needle electrodes suggests they may be preferable for future HFO studies when clinically feasible. Although these comparisons are exploratory and potentially confounded by recording context, needle electrodes appear advantageous for research‐grade HFO acquisition when safety and logistics permit.

Given prior animal work^47^, ^48^, ^68^, ^69^ suggesting that pHFOs are more prominent over pericontusional tissue and our observations, electrode strategy may matter. When clinically indicated, subdural strip coverage of pericontusional cortex could increase pHFO detection sensitivity relative to depth‐only sampling, which often interrogates radiographically normal‐appearing tissue. This is also consistent with scalp studies in lesional cohorts, where greater structural injury was associated with higher scalp HFO rates and broader fields.^75^, ^76^, ^77^

To our knowledge, this is the first study to systematically examine the relationship between HFOs and late PTS in patients with acute hemorrhagic TBI, and the first to assess the detectability of fast ripples on scalp EEG in this population using the routine clinical EEG recording, where the main adjustment was using a higher sampling rate. Despite the inherent difficulties of detecting HFOs in critically ill patients where EEG is often contaminated by artifacts, we were able to observe ripples in some patients using high sampling rates (≥500 Hz for ripples and ≥1000 Hz for fast ripples) and rigorous preprocessing as well as thorough artifact rejection.

### 4.1 Limitations

The limitations of our study can be summarized as follows: (1) This is a relatively small cohort and (2) EEG technical limitations of sampling rate and electrode placement likely contributed to the absence of verified detection of fast ripples. Future studies will benefit from standardized acquisition protocols, sampling rates matched to the target band (≥2000 Hz), and rigorous artifact rejection. Prospective tracking of sedatives and other interventions would further minimize confounding, potentially through novel monitoring systems enabling automatic real‐time logging of medications administered. (3) Duration of monitoring was likely too short to detect subacute development of pHFOs in the days to weeks following TBI, hence our window of observation may be too early to appreciate the development of pHFOs. (4) Lesion location and size heterogeneity likely influences the development of pHFOs and the proximity of surface EEG electrode location to pHFO generator sites. (5) Automated methods for pHFOs require human validation, and hence there is possible error related to interrater reliability during the validation step.

## 5 CONCLUSIONS

To the best of our knowledge, this is the first study to investigate the relationship between pHFOs and the development of late PTS in a human cohort with acute hemorrhagic TBI.

In our cohort, verified pHFOs were most frequently observed over frontal and perihemorrhagic regions, suggesting that these areas may be particularly prone to early postinjury hyperexcitability. These data suggest partial validation of preclinical observations regarding pHFOs during epileptogenesis in the human TBI condition.

## AUTHOR CONTRIBUTIONS

Kseniia Kriukova, Richard J. Staba, Jerome Engel, Paul M. Vespa, and Dominique Duncan conceived the study. Kseniia Kriukova, Lin Li, Richard J. Staba, Mohamad Shamas, Jerome Engel, and Paul M. Vespa developed the methodology. Kseniia Kriukova, Manuel Buitrago Blanco, and Paul M. Vespa curated the data. Kseniia Kriukova, Manuel Buitrago Blanco, Jerome Engel, Paul M. Vespa, and Dominique Duncan carried out the investigation. Kseniia Kriukova, Lin Li, Richard J. Staba, James J. Gugger, Rachel Thomas, Ramon Diaz‐Arrastia, and Mohamad Shamas performed validation. Kseniia Kriukova, Lin Li, Richard J. Staba, Mohamad Shamas, Jerome Engel, Paul M. Vespa, Rachel Thomas, Manuel Buitrago Blanco, Misque Boswell, Tuba Asifriyaz, James J. Gugger, Ramon Diaz‐Arrastia, and Dominique Duncan contributed to writing—review & editing. Richard J. Staba, Ramon Diaz‐Arrastia, Jerome Engel, Paul M. Vespa, and Dominique Duncan provided supervision. Jerome Engel, Paul M. Vespa, and Dominique Duncan secured funding.

## FUNDING INFORMATION

J.E. is supported in part by NINDS RF1 NS033310 and the Christina Louise George Trust. R.J.S. is supported in part by NINDS U54 NS100064, R01 NS106957, RF1 NS033310, and R01 NS127524 and the Christina Louise George Trust. P.M.V. is supported by NINDS U54 NS100064. D.D. is supported by NINDS U54 NS100064.

## CONFLICT OF INTEREST STATEMENT

All authors have no conflicts of interest to disclose. We confirm that we have read the Journal's position on issues involved in ethical publication and affirm that this report is consistent with those guidelines.

## Data Availability

The data that support the findings of this study are available from Data Archive for the BRAIN Initiative (https://dabi.loni.usc.edu). Restrictions apply to the availability of these data.
